# Supplementary material for: Cognitive control of behavior and hippocampal information processing without medial prefrontal cortex
Source: eLife. 2025 Jun 23;13:RP104475. doi: 10.7554/eLife.104475 (PMC12185103; doi:10.7554/eLife.104475)
Supplement: Supplementary file 1. — Relative cytochrome oxidase (CO) activity/μm tissue (× 10–1). RSD, dysgranular retrosplenial cortex; RSG, granular retrosplenial cortex; RE, the nucleus reuniens; CEA, the central nucleus of the amygdala; BMA, basomedial amygdala; BLA, basolateral amygdala; dCA1, dorsal CA1; dCA2, dorsal CA2; dCA3, dorsal CA3; dDG, dorsal dentate gyrus; vCA1, ventral CA1; vCA3, ventral CA3; vDG, ventral dentate gyrus; DS, dorsal subiculum (sham; n=8, lesion; n=8). [file elife-104475-supp1.docx]

| Brain Region | Sham  (Avg. ± SEM) | Lesion  (Avg. ± SEM) | t value | *p-value* |
| --- | --- | --- | --- | --- |
| RSD | 1.84 ± 0.09 | 2.01 ± 0.14 | 1.04 | 0.32 |
| RSG | 1.91 ± 0.09 | 2.16 ± 0.09 | 1.86 | 0.08 |
| RE | 1.47 ± 0.08 | 1.66 ± 0.09 | 1.51 | 0.15 |
| CEA | 1.27 ± 0.18 | 1.94 ± 0.19 | 2.61 | **0.02** |
| BMA | 1.31 ± 0.11 | 1.54 ± 0.20 | 0.99 | 0.34 |
| BLA | 1.33 ± 0.17 | 1.72 ± 0.20 | 1.49 | 0.16 |
| dCA1 | 1.51 ± 0.08 | 1.60 ± 0.04 | 0.97 | 0.35 |
| dCA2 | 1.34 ± 0.08 | 1.43 ± 0.06 | 0.98 | 0.34 |
| dCA3 | 1.50 ± 0.10 | 1.63 ± 0.10 | 0.94 | 0.36 |
| dDG | 1.39 ± 0.08 | 1.50 ± 0.10 | 0.85 | 0.41 |
| vCA1 | 1.54 ± 0.15 | 1.74 ± 0.14 | 1.02 | 0.33 |
| vCA3 | 1.73 ± 0.14 | 1.92 ± 0.15 | 0.94 | 0.36 |
| vDG | 1.75 ± 0.12 | 1.92 ± 0.13 | 0.92 | 0.37 |
| DS | 1.36 ± 0.14 | 1.61 ± 0.19 | 1.03 | 0.32 |
