# Supplementary material for: Cognitive control of behavior and hippocampal information processing without medial prefrontal cortex
Source: eLife. 2025 Jun 23;13:RP104475. doi: 10.7554/eLife.104475 (PMC12185103; doi:10.7554/eLife.104475)
Supplement: Supplementary file 2. [file elife-104475-supp2.docx]

|  |  | **Time to First Enter** | | | |  | **Entrances** | | | |
| --- | --- | --- | --- | --- | --- | --- | --- | --- | --- | --- |
| **Rat** | **Cells** | **Pretrain** | **Initial** | **Retention** | **Conflict** |  | **Pretrain** | **Initial** | **Retention** | **Conflict** |
| mPFC01 | 17 | 17.9 | 315.2 | 419.4 | 139.7 |  | 14.5 | 2.7 | 1.0 | 8.6 |
| mPFCS02 | 70 | 10.3 | 128.4 | 103.7 | 58.5 |  | 16.0 | 6.8 | 4.0 | 7.4 |
| mPFC3 | 15 | 2.9 | 251.8 | 14.1 | 39.5 |  | 16.0 | 5.9 | 12.0 | 14.3 |
| mPFC04 | 15 | 77.3 | 169.7 | 107.1 | 117.6 |  | 12.0 | 5.7 | 2.0 | 11.5 |
| mPFC07 | 24 | 0.8 | 99.9 | 32.9 | 30.0 |  | 16.0 | 10.8 | 6.0 | 10.8 |
| mPFC08 | 46 | 29.3 | 135.0 | 84.1 | 349.5 |  | 22.5 | 4.4 | 11.0 | 5.5 |
