# Supplementary material for: Cognitive control of behavior and hippocampal information processing without medial prefrontal cortex
Source: eLife. 2025 Jun 23;13:RP104475. doi: 10.7554/eLife.104475 (PMC12185103; doi:10.7554/eLife.104475)
Supplement: Supplementary file 3. [file elife-104475-supp3.docx]

| **Thalamus (187 cells)** | **Sham lesion** | **mPFC Lesion** | **t(d.f); p values** |
| --- | --- | --- | --- |
| Firing rate (AP/s) | 14.81 ± 1.74 | 19.00± 2.16 | 1.51(183); 0.13 |
| Burst ratio | 1.08 ± 0.07 | 0.76 ± 0.08 | 2.91(174); 0.004 |
